# Living alone is a risk factor for mortality in men but not women from the general population: a prospective cohort study

**DOI:** 10.1186/1471-2458-7-335

**Published:** 2007-11-16

**Authors:** Ulla Kandler, Christa Meisinger, Jens Baumert, Hannelore Löwel, the KORA Study Group

**Affiliations:** 1 GSF-National Research Center for Environment and Health, Institute of Epidemiology, Ingolstädter Landstr. 1, 85764 Neuherberg, Germany.; 2 Central Hospital of Augsburg, MONICA/KORA Myocardial Infarction Registry, Stenglinstr. 2, 86156 Augsburg, Germany.

## Abstract

**Background:**

During the past decades a rising trend of living alone can be observed in the population especially in urban areas. Living alone is considered a psychosocial risk factor. We studied the relationship between living alone, cardiovascular risk factors and mortality.

**Methods:**

We analysed data from the population-based MONICA/KORA cohort study including 3596 men and 3420 women of at least one of three surveys carried out between 1984 and 1995 in the region of Augsburg, Germany. They were between 45 and 74 years old and were followed-up until 31 December 2002. During follow-up 811 men and 388 women died. Cox proportional hazards analysis was used to examine the association between living alone and mortality.

**Results:**

Altogether 260 men (7%) and 620 women (18%) were living alone at baseline. Men, who lived alone, were less well educated, had fewer children and friends, and they smoked significantly more than other men. Women, living alone, were also significantly more often current smokers and had less children and friends, but they were more often better educated than cohabitating women. The latter group showed a higher proportion of obese and hypertensive women. Men living alone had a twofold risk to die after multivariable adjustment (hazard ratio = 1.96; p < 0.0001; 95% confidence interval 1.56–2.46). This was not the case for women.

**Conclusion:**

Living alone is an independent risk factor for mortality in men. It is unclear whether living alone causes an increased mortality or whether predisposition for increased mortality is responsible for men living alone.

## Background

According to the German micro-census 2004, almost 40 percent of the households are so-called "single-person households" (Statistisches Bundesamt 2005). They are defined as households in which one person is living alone. Living alone is often equated with social isolation, which is supposed to be associated with an increased risk of mortality [1-7]. Recently, Schmaltz et al. used living alone as a proxy for social isolation and found an increased post-acute myocardial infarction mortality for persons who live alone. Kharicha et al. reported a significant association between living alone and the risk of social isolation, when they examined the clinical significance of living alone [8]. Routasalo et al investigated the relationship between emotional loneliness and social isolation among the older Finnish population and found that living alone was a strong predictor for loneliness [9]. Thus living alone may be a valuable tool to assess social isolation or emotional loneliness, which are both associated with increased morbidity and mortality.

However, the pathway of this association is not completely clear. There are at least two possible mechanisms: Psychosocial factors may influence a person's health behaviour and/or they may have direct effects on patho-physiological processes [10,11]. People, who live alone, form a very heterogeneous group though. The diversity ranges from those, who are socially and professionally disintegrated, mostly men, to those, in the majority of cases women, who are well educated and socially and professionally successful [12]. Living alone must also be seen in the context of age. For young people, living alone is associated with the period of life, where they have left their home of origin and have not yet founded their own family or partnership. For older persons, living alone often is the result of widowhood or divorce. Since single-person households are very common [Stat. Bundesamt 2005] an association between living alone and mortality may have great public health implications. In the present study, we examined the association between living alone and mortality for 45- to 74-year old persons who were followed up for over ten years in the region of Augsburg, Southern Germany. Because we hypothesized that living alone does not have the same predictive impact among men and women, gender-specific analyses were conducted.

## Methods

### Study design and study population

The present analysis was performed with data from the three MONICA (Monitoring trends and determinants on cardiovascular disease) Augsburg (Southern Germany) surveys, which were carried out in 1984/85, 1989/90 and in 1994/95 to assess the prevalence of cardiovascular risk factors in the general population. These cross-sectional surveys were based on randomly sampled individuals from the city of Augsburg and the counties of Augsburg and Aichach/Friedberg. Details of the MONICA Augsburg project have been described elsewhere [13-16].

A total number of 13 427 persons aged 25 to 74 years at baseline participated in at least one of the three surveys (6725 men, 6702 women, response 77%) and were followed prospectively through the Cooperative Health Research in the Region of Augsburg (KORA). Persons who had participated in more than one survey, were included only once in the analysis with the data from their first survey.

### Inclusion/exclusion criteria

All persons between 25 to 44 years at baseline (2732 men and 2872 women) were excluded. Individuals with incomplete information on relevant variables were also excluded from the analysis (397 men and 409 women). The final sample consisted of 3596 men and 3421 women aged 45 to 74 years at baseline. All persons had given written informed consent and the study was approved by the local ethics committee.

### Data collection

Data collection at baseline was done by a standardized interview and a clinical examination by trained medical staff and a self-administered questionnaire. Socio-demographic variables like years of education, marital status, occupation and place of living were obtained through the interview. The participants were also asked to provide details on their health behaviour in regards to smoking, alcohol consumption, physical activity and nutrition as well as health care utilization. Chronic diseases and current medication were assessed. All study participants rated their current health status as either excellent, good, fair or poor. Physical examination comprised measurements of blood pressure, weight and height. A non-fasting venous blood sample was drawn from the participants. The methods of measurement have been described elsewhere [15,16].

### Definitions and formation of variables

Hypertension was defined as a blood pressure of 140/90 mmHg and higher, and/or use of antihypertensive medication, given that the subjects were aware of being hypertensive. The body mass index (BMI) was calculated and divided into three groups: <25, ≥ 25 and <30, and ≥ 30 kg/m^2 ^corresponding to normal weight, overweight and obesity, respectively. The total cholesterol to HDL cholesterol ratio was calculated. A ratio of 5 or higher was considered as potentially pathogenic. Participants were classified as current smokers, ex-smokers or non-smokers. Daily alcohol consumption was divided into three groups (none, 0–20 g/d for women, 0–40 g/d for men, >20 g/d for women and > 40 g/d for men). Individuals were considered as physically active during leisure time, if they performed any sport one or more hours per week in summer and winter. The self-rated health variable was dichotomized by combining the categories "excellent" and "good" on the one hand, and "fair" and "poor" on the other hand.

### Assessment of living alone and social relationships

Whether a person lived alone was established by questioning if there were smoking cohabitants in the household. One possibility to answer was: "I am living alone in my household". This answer was used to create the dichotomous variable "living alone" (yes/no). Living alone is defined as a person living alone in his/her household, independent from marital status, the number of children, friends and/or relatives.

The marital status of participants was assessed separately through a question in the standardized interview. The corresponding variable was called "marital status" and included the categories single, married, separated and widowed.

The self-administered questionnaire focused on psychosocial questions concerning the daily life of each person, asking whether they had children and social relationships with friends and relatives.

We divided the participants into two groups regarding the number of friends and/or relatives they reported to have. Those with 11 or more friends and relatives were classified "many friends/relatives", persons with 10 or less friends/relatives received the label "few friends/relatives". We chose10 as cut-off as the two questions on number of relatives and number of friends is originally combined on a four point scale (1:0–5, 2:6–10, 3:11–15, 4: >15 friends or relatives) which was later transformed into a two point scale (1:0–10, 2: >10).

### Outcome

The vital status of all participants was regularly checked through the population registries in- and outside the study area. Death certificates were obtained from local health departments and coded for the underlying cause of death by a single trained person using the 9^th ^revision of the International Classification of Diseases (ICD-9).

### Statistical analysis

Sex-specific and age-adjusted prevalences of all parameters were calculated and logistic regression was used to compare these prevalences between persons living alone and those living not alone.

The duration of follow-up was derived from the difference between the date of baseline examination and the date of death or last follow-up information until 31^st ^December 2002. Crude sex-specific all cause mortality as well as cardiovascular disease mortality rates were calculated for the whole study sample per 1000 person years (pyrs).

The subsequent analyses were stratified by sex. The association between living alone and mortality was examined using Cox proportional hazards models. The assumption of proportional hazards was tested and fulfilled. In a first step, the crude association between living alone and mortality was calculated (Model 1). The second model adjusted for age (continuous) and survey (three categories for the three surveys). We consecutively adjusted for socio-demographic variables (Model 3), classical cardiovascular risk factors (Model 4) and indicators of health behaviour (Model 5). Socio-demographic variables were the reporting unit (living in the urban area of the study area), years of education, having children and contact with friends and relatives. Classical cardiovascular risk factors were represented through history of diabetes and/or myocardial infarction, hypertension, angina pectoris, hypercholesterolemia, obesity and self-rated health. Indicators of health behaviour were on the one hand physician and dentist visits and participation in a cancer screening program during the year before participating in the study as a measure of health care utilization and on the other hand smoking, alcohol consumption and physical activity. A final model was built using all variables which had a significant effect on mortality at the 10% level in the last model.

Else significance tests were two-tailed and p-values less than 0.05 are stated as statistically significant. All analyses were performed using the Statistical Analysis System (Version 8.02, SAS Institute Inc., Cary, NC).

## Results

### Description of the study population

**Figure 1:**
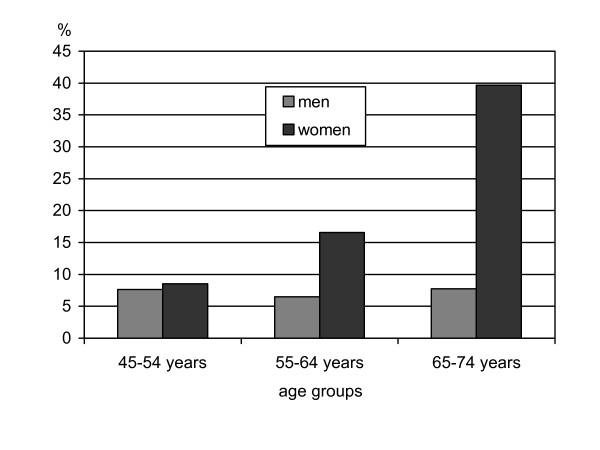
Proportion of men and women who live alone per age group.

At baseline, 620 (18%) women and 260 (7%) men were living alone. According to the micro census of the Augsburg region 17% of all households belonged to alone living women and 11% to alone living men in the age group of 45 to 75 year old persons. In our study, the age distribution differed significantly (p-value ≤ 0.0001) between the group of women living alone and the other women; this was not the case in the male subgroups. Figure 1 provides the frequencies of living alone by sex and age group. Women in the age-group between 65 and 74 were more likely to live alone than younger female participants. The mean follow-up time was 11.4 years with a range from 0.1 to 18.2 years. The total number of person years of the 7017 participants added up to 80341.8 years.

Table 1 and 2 show the age-adjusted prevalences for persons, living alone and who do not live alone for socio-demographic variables, classical cardiovascular risk factors and health behaviours. In summary, the men living alone were more likely to be current smokers, they had shorter education times and had significantly fewer children and friends.

**Table 1:** Age-adjusted prevalence of baseline characteristics in men (n = 3596)

**Characteristics**	**Not living alone**			**Living alone**			
**Men**	Prevalence*	**95% C.I.**		Prevalence*	**95% C.I.**		**P-value**
***Sociodemography***							
Living in urban area	45.7	44.0	47.4	51.5	45.5	57.6	0.07
Education >= 12 years	30.5	29.0	32.1	23.7	18.9	29.2	0.02
Children	88.0	86.8	89.0	55.4	49.3	61.3	<0.0001
Friends/relatives > 10	29.5	28.0	31.1	21.9	17.3	27.3	0.01
***Risk factors/comorbidities***							
Hypertension	55.9	54.2	57.6	54.0	47.8	60.1	0.56
HDL/total Cholesterol >= 5	49.3	47.6	50.9	48.5	42.4	54.5	0.81
Prevalent myocardial infarction	4.3	3.6	5.1	4.2	2.4	7.1	0.93
Prevalent diabetes	7.1	6.3	8.1	8.1	5.4	12.0	0.54
body mass index <25	19.3	18.0	20.7	27.3	22.2	33.0	0.002
25 <= body mass index < 30	57.9	56.2	59.6	50.4	44.3	56.4	0.02
body mass index >= 30	22.7	21.3	24.2	22.2	17.6	27.7	0.85
Angina pectoris	5.3	4.6	6.2	6.7	4.2	10.4	0.36
Unfavourable self rated health	26.2	24.7	27.7	29.5	24.3	35.3	0.25
***Health behaviours***							
Physician visit (last 12 months)	84.7	83.3	85.9	84.8	80.0	88.6	0.95
Cancer screening (last 12 months)	23.0	21.6	24.5	21.7	17.1	27.2	0.63
Dentist visit (last 12 months)	64.0	62.3	65.6	62.9	56.8	68.6	0.73
Physically active	37.8	36.2	39.5	34.8	29.3	40.9	0.34
Current smoker	24.0	22.6	25.5	37.4	31.6	43.5	<0.0001
Alcohol >= 40 g/d	31.8	30.2	33.4	33.9	28.4	40.0	0.48

**Table 2:** Age-adjusted prevalence of baseline characteristics in women (n = 3421)

**Characteristics**	**Not living alone**			**Living alone**			
**Women**	Prevalence*	**95% C.I.**		Prevalence*	**95% C.I.**		**P-value**
***Sociodemography***							
Living in urban area	42.5	40.7	44.4	54.8	50.7	58.8	<0.0001
Education >= 12 years	10.7	9.6	12.0	16.1	13.2	19.6	0.0007
Children	87.7	86.4	88.9	70.3	66.3	74.0	<0.0001
Friends/relatives > 10	30.5	28.8	32.2	25.8	22.4	29.6	0.03
***Risk factors/comorbidities***							
Hypertension	48.6	46.7	50.6	42.6	38.4	46.8	0.01
HDL/total Cholesterol >= 5	25.7	24.1	27.4	23.9	20.6	27.4	0.34
Prevalent myocardial infarction	0.9	0.6	1.4	1.1	0.5	2.1	0.65
Prevalent diabetes	4.7	3.9	5.5	4.7	3.4	6.5	0.90
Body mass index < 25	28.8	27.1	30.6	35.2	31.2	39.4	0.005
25 <= body mass index < 30	42.5	40.7	44.4	41.1	37.1	45.2	0.54
Body mass index >= 30	27.6	26	29.4	23.3	20.1	26.9	0.03
Angina pectoris	4.5	3.8	5.3	4.9	3.4	6.9	0.67
Unfavourable self rated health	32.8	31.1	34.6	34	30.3	38.0	0.58
***Health behaviours***							
Physician visit (last 12 months)	89.9	88.7	91.0	93.3	90.9	95.1	0.02
Cancer screening (last 12 months)	44.4	42.5	46.3	44.5	40.3	48.8	0.97
Dentist visit (last 12 months)	67.6	65.8	69.3	67.5	63.5	71.2	0.96
Physically active	30.5	28.8	32.3	33.9	30.1	38.0	0.12
Current smoker	11.1	9.9	12.3	21.8	18.3	25.7	<0.0001
Alcohol >= 20 g/d	16.4	15	17.8	17.7	14.7	21.1	0.47

**Figure 2:**
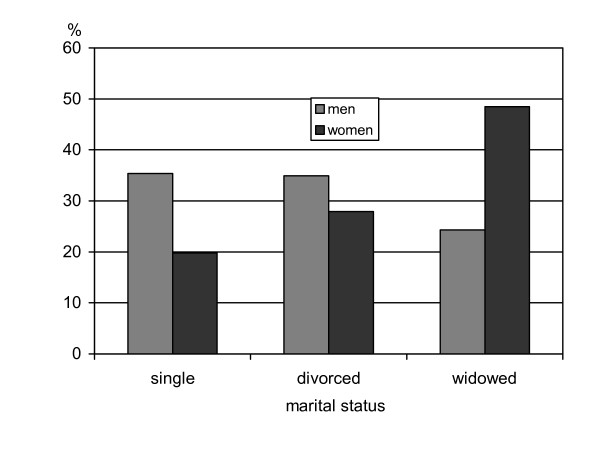
Marital status of men and women who live alone.

Women, who lived alone, were also more likely to be current smokers than cohabitating women. They were more often non-obese and less likely to suffer from hypertension. Further, they consulted their physicians more often, had enjoyed longer education times and lived in urban areas. They had less children and friends/relatives than their counterparts, who lived with somebody. Figure 2 shows the age-adjusted prevalence of marital status of men and women living alone. While the percentage of men who were single, divorced or widowed remained relatively constant at around 30%, the rates in women varied considerably, with fewer being single (20%), almost the same percentage being divorced (27%), and by far more being widowed (49%).

### Mortality

By December 31, 2002, 1031 men and 520 women had died. Table 3 presents the crude mortality rates for each sex by age group and cause of death.

**Table 3:** Crude mortality rates by sex and age group

	**Not living alone**				**Living alone**			
**Age in years**	**total N of deaths**	Total mortality*	**N of CVD deaths**	CVD mortality*	**total N of deaths**	Total mortality*	**N of CVD deaths**	CVD mortality*
**Men**								
45 – 54 y.	132	8.5	50	3.3	12	11.2	5	3.8
55 – 64 y.	333	22.9	144	10.1	32	37.7	14	17.8
65 – 74 y.	260	43.0	123	21.0	42	90.0	19	42.3
All	725	20.5	317	9.3	86	35.3	38	15.9
**Women**								
45 – 54 y.	61	4.0	17	1.3	2	2.0	0	0.0
55 – 64 y.	147	11.4	66	5.1	29	11.1	14	4.7
65 – 74 y.	77	21.7	35	9.1	72	32.7	30	13.2
All	285	9.2	118	3.8	103	18.4	44	7.37

*per 1000 person years

The hazard ratio (HR) for all cause mortality was 2.05 (95% confidence interval (CI) 1.66–2.60) for men living alone and 1.13 (95% CI 0.89–1.44) for women living alone after adjusting for age and survey (Model 2). Subsequent adjustments for socio-demographic factors (Model 3) and for cardiovascular risk factors (Model 4) showed remaining significant associations in men (HR = 1.97, 95% CI 1.6–2.48 Model 3 and HR = 2.07, 95% CI 1.64–2.61 Model 4) and non-significant associations in women (HR = 1.10, 95% CI 0.86–1.40 Model 3 and HR 1.10, 95% CI 0.84–1.37 Model 4). Further adjustment for health behaviour (smoking, alcohol consumption, physical activity, physician and dentist visits and participation in cancer screening) only slightly attenuated the associations (Model 5: men: HR = 1.87, 95% CI 1.48–2.37 and women: HR = 1.03, 95% CI 0.80–1.32). In the final model the HR for living alone was 1.96 (95% CI 1.56–2.46) for men and 1.11 (95% CI 0.87–1.41) for women (Table 4; final model). The HR for cardiovascular disease mortality was 2.00 (95% CI 1.41–2.82) for men living alone and 1.12 (95% CI 0.67–1.48) for women living alone after multivariable adjustment in the final model (Table 4; final model).

**Table 4:** Gender-specific hazard ratios and 95% C.I. for all cause and CVD mortality according to living alone at baseline

**Mortality**	Hazard Ratio**	**95% C.I.**		**P-value**	Hazard Ratio***	**95% C.I.**		**P-value**
**Men**								
Total mortality*	2.07	1.66	2.60	<0.0001	1.96	1.56	2.46	<0.0001
Cardiovascular mortality*	2.13	1.52	2.99	<0.0001	2.00	1.41	2.83	<0.0001

**Women**								
Total mortality*	1.12	0.88	1.42	0.36	1.11	0.87	1.41	0.41
Cardiovascular mortality*	1.06	0.73	1.53	0.77	1.02	0.70	1.48	0.94

* compared for persons who live alone with those who don't after multivariable adjustment

** adjusted for age and survey

*** final model men: adjusted for age, survey, number of friends, prevalent MI and diabetes, hypertension, self rated health, obesity, participation in screening, dentist visits, physical activity, alcohol consumption, smoking

final model women: adjusted for age, survey, prevalent diabetes, hypertension, self rated health, participation in screening, dentist visits, physical activity, smoking

### Missing variables

The group of persons, excluded from the analysis due to missing values, mainly concerning the variables 'children' and 'friends', was compared to the study subjects with regard to sex, age, education and number of deaths. While in the excluded group more subjects were older or died, educational levels and sex distribution were approximately the same in both groups.

We subsequently constructed a Cox model that included the formerly excluded individuals and did not adjust for 'children' and 'friends'. The multivariable adjusted HR of total mortality in men and women who lived alone was 1.73 (95% CI 1.41–2.13) and 1.07 (95% CI 0.87–1.32), respectively, which was generally similar to the presented results.

## Discussion

The present study found that living alone was a significant predictor of mortality for middle-aged men, but not for women, from the general population. Men who live alone have about twice the risk to die from all causes and cardiovascular diseases, independent of cardiovascular disease risk factors, health behaviour, co-morbidities, and socio-demographic factors. Other studies have demonstrated that marital status and social integration have strong effects on mortality in men [1,17-19], in women only [20] or in both sexes [2,19,21,22]. Although cohabitation status is associated with marital status and/or social integration, the agreement between these three items is not complete. Lund et al. [23] found a stronger effect of living alone than of marital status as a predictor of mortality among men and women aged 50, 60, and 70 years. They therefore suggested that cohabitation status be considered a predictor of mortality in future studies rather than marital status. Contrary to our study, they demonstrated strong effects of cohabitation status on mortality in both women and men without age differences in the association. Furthermore they found that adjusting for health behaviour, including smoking, diet, and physical activity, did not change the association between living arrangements and mortality, thus leading them to dismiss changes in health behaviour due to cohabitation status as one possible explanation for the difference in mortality between those who do not live alone and those who do. The latter findings, however, were supported by our study, as we also found that adjusting for the health behaviour factors smoking, alcohol intake, and physical activity only slightly attenuated the effect of living alone on mortality in men. In contrast to the study of Lund et al., in the present study even more detailed information on each participant was available, which we included in the Cox models without observing any significant changes of the main effect. Davis and Moritz [24] examined the effect of living arrangements in a cohort of elderly persons from the Longitudinal Study of Aging from 1984 to 1990 and found no association between living alone and mortality. They argue that a necessary prerequisite for living alone is good health and adequate coping strategies for daily living. The age group they examined was 70 years and above whereas our study population was between 45 and 74 years old, which might explain the differing findings. No stratification by age group was conducted in the present study because of limited number of participants, particularly in men, to test this explanation. In our study the percentage of women aged 45–74 years who lived alone (18%) was much higher than the percentage of men (7%), which is in accordance with publications from other authors [1,17,18]. In the present study, the proportion of women living alone increased with age, whereas the proportion of men, living alone, remained constant between 45 and 74 years. This may be explained by the increased risk of widowhood as women age, causing a shift from the group with cohabitants to the group of those living alone. This shift is not detectable in men. An analysis of the marital status in men and women living alone confirmed this hypothesis. The age-adjusted prevalence of single women is lower than the percentage of single men, whereas the percentage of widowed women is twice the percentage of widowed men. The percentage of single, divorced or widowed persons in the cohabitating groups are comparatively low (5% of men, 12% of women), but not negligible. It becomes clear that marital status is not the same as cohabitation status and therefore should always be considered separately. The group of women living alone not only differed from the corresponding group of men with regards to age distribution and marital status, but also in education times. Although the percentage of women with longer education times was lower compared to men, it was higher compared to cohabitating women. Men with education times over 12 years were more frequently found in the cohabitating group. These findings confirm the statements from Streuli's and Höpflinger's essay [12], that the group of persons who live alone is heterogeneous. Furthermore, it becomes clear that the equation of living alone with social isolation needs to be treated with care, because living alone does not have the same effect on women as on men.

Further studies are needed to elucidate what impact living alone has on men and to assess the reasons for their living alone. Differences in mental and other pre-existing diseases or in health behaviour might explain the association between living alone and mortality. However, our data do not suffice to answer these questions.

Other limitations of the MONICA/KORA Augsburg cohort study need to be considered. Since the question of whether a person lived alone or not was asked at a certain point in time, we cannot say if this was a permanent or temporary condition and how long it lasted. In addition the exposure living alone is not measured directly, because it was asked in the context of the assessment of smoking habits in the standardized interview. But in spite of it's differing purpose, the question was answered well with only few missing values. The information was very plausible and can thus be considered reliable. Information on dietary habits and depression was incomplete and, therefore, was not included in the analysis. Furthermore, there were no measures of physical functioning available. Hence residual confounding cannot entirely be excluded. Because the study was limited to 45–74-year-old men and women of German nationality, caution should be used in generalizing these results to other ethnicities and age-groups. Participants of the MONICA/KORA cohort study were likely to be younger, healthier, and better educated than non-participants, which might have introduced a selection bias [25,26]. Compared to the general population of the Augsburg region less men living alone participated in the study, which might also cause selection bias. The strengths of the MONICA/KORA Augsburg Cohort Study are primarily its prospective design, long follow-up times, the representativeness of the cohort, being based on a random sample of the general population, the inclusion of hard endpoints, and the availability of standardized data on life style and multiple cardiovascular risk factors.

## Conclusion

Living alone was identified as an independent risk factor for total and cardiovascular mortality in 45 to 74 year old men, but not in a corresponding group of women from the general population. Cohabitation status is an easily assessed parameter in studies and has been shown to make a meaningful contribution to an analysis of mortality. Caution is warranted because the group of persons living alone is heterogeneous and living alone does not have the same consequences for every individual. Thus, it cannot replace the assessment of social relations. Future research will have to focus on determining the reasons for, and the burdens of, living alone, especially as it relates to the male population.

## Competing interests

The author(s) declare that they have no competing interests.

## Authors' contributions

UK contributed to the conception of the paper, analysed and interpreted the data and drafted the manuscript. HL and CM contributed to the conception of the paper and the interpretation of the data. JB was involved in the statistical analysis. All authors read and approved the final manuscript.

## Pre-publication history

The pre-publication history for this paper can be accessed here:


